# The Street-Cars a Menace to Public Health

**Published:** 1889-03

**Authors:** 


### *THE STREET-CARS A MENACE TO PUBLIC HEALTH.*

The Board of Health cannot expect to abate contagious diseases, such, for instance, as small-pox, which has been lurking in our midst for some months, (though, happily, now nearly stamped out through the energetic action of the health authorities,) until it sets itself vigorously at work to abate the street-car nuisance, which is an ever-present carrier of contagion under the existing conditions.

Nor is it alone infectious diseases that this atrocious system tends to foster or propagate. Let us speak specifically: Starting at the foot of Main street, or at one of the railroad stations, one of these frosty mornings, the traveler is ushered into a car somewhat generously carpeted with straw or hay. Before he reaches the business centers, he will be fortunate if he is not attacked with an irritating cough, caused by the particles of dust arising from the trampled hay or straw, which he must perforce inhale; and he will be still more fortunate if his ears and finger tips are not frozen stiff by the time he reaches Music Hall. There can be no doubt that this is one of the easy methods to contract a pneumonia, to say nothing of the multitude of other diseases that may be "taken" in this manner.

Attention has been called in the secular press to the unhealthfulness of this method of transit, provided by the large-hearted and public-spirited Buffalo Street Railway Company, time and time again, without avail, and we now feel it our duty to invite an official investigation of this matter by the Board of Health, which, as the natural custodian of all questions involving sanitation, must take cognizance thereof; nor shall we be content until the nuisance complained of is abated.

We base our complaint entirely upon public health grounds, letting alone the questions of propriety and common decency in reference to the management of the street railway system. Every intelligent woman knows that she forfeits her self-respect when she enters one of these disease-breeding and conveying vehicles, and she does so under mental protest. The contrast between a well-warmed, cleanly car, such as may be seen in most other cities, and one of these, ought to bring shame to the owners of the franchise; but when the question of public health becomes involved, it is a most serious indictment, and merits the thoughtful consideration of every citizen who has the interests of the municipality at heart, as well as humanitarians, sanitarians, public health officials, and even the constituted judicial authorities. It is puerile, in the present state of science, to say that a properly warmed car cannot be adequately ventilated. This is just the sort of car, above all others, that can be properly ventilated.

For ourselves, we refrain from riding in the street-cars in their present unhealthy state, nor do we expect to avail ourselves of this privilege until they are properly warmed in Winter and kept in a cleanly condition. Furthermore, we advise our readers to avoid them if they would seek health and longevity.

We appeal to the Board of Health to take prompt and energetic action in this matter. Nothing but the strong arm of the law, which is framed to protect the citizen in the enjoyment of health and prosperity, can reach this already deeply-rooted and, we fear, conscience-seared nuisance.

